# On the Nerves of the Bone

**Published:** 1853-01

**Authors:** M. Koelliker


From the Provincial Medical and Surgical Jour.

*On the Nerves of the Bone, by* M. Koelliker.

M. Koelliker states that, in the human subject, the nerves accompany the blood-vessels not only in the medulla of the long bones, but also in the spongy tissue of the epiphyses. The short bones, such as the vertebræ, are richly supplied with nervous filaments, as are also the blade-bones and ilia. They are also readily demonstrated in the bones of the cranium. The function of these nerves he considers to be chiefly that of nutrition; but that they contain sensitive fibres is plainly shown by the pain which attends disease of osseous structure.

